# Peripartum Complications as Risk Factors for Postpartum Psychosis: A Systemic Review

**DOI:** 10.7759/cureus.29224

**Published:** 2022-09-16

**Authors:** Kim Nguyen, Lawrance T Mukona, Linette Nalbandyan, Nadia Yar, Guinda St. Fleur, Lorraine Mukona, Edward Hernandez, Norman Lamberty

**Affiliations:** 1 Obstetrics and Gynecology, American University of Antigua, Osbourn, ATG; 2 Psychiatry, American University of Antigua, Osbourn, ATG; 3 College of Medicine, American University of Antigua, Osbourn, ATG; 4 Internal Medicine, American University of Antigua, Osbourn, ATG; 5 Obstetrics and Gynecology, Montclair Medical Center, Montclair, USA

**Keywords:** pre-eclampsia, cesarian section, periparturient complications, postpartum psychosis risk factors, postpartum psychosis etiology, postpartum psychosis

## Abstract

The aim of this research paper is to conduct a systematic review of periparturient complications as risk factors of postpartum psychosis. The investigation of risk factors for maternal psychosis following childbirth is complicated by the risk of confounding by a previous psychiatric history; therefore, this systematic review focuses on labor complications as risk factors among women without any previous psychiatric hospitalizations or diagnoses.

Articles were collected and analyzed from the PubMed, MEDLINE, and Cochrane Review Library databases, as well as Clinicaltrials.gov, in accordance with the 2020 Preferred Reporting Items for Systematic Reviews and Meta-Analyses (PRISMA) guidelines. Article abstracts and article titles of the identified publications were screened independently by all seven authors, and studies were selected if they met the following inclusion criteria: patients were diagnosed with postpartum psychosis per the guidelines in the American Psychiatric Association’s Diagnostic and Statistical Manual of Mental Disorders (DSM-V), DSM-IV or World Health Organization’s ICD-10 Classification of Mental and Behavioral Disorders; patients presented with no prior psychiatric diagnoses, hospitalizations or history; and the study evaluated the association of periparturient complications to first-onset postpartum psychosis, excluding narrative reviews, systematic reviews, or meta-analyses.

Fifteen case-control, cohort, and case report studies, with thousands of patients, were selected to investigate the correlation between perinatal complications and first-onset post-partum psychosis. Obstetric complications during childbirth significantly predisposed for postpartum psychosis in certain individual studies but did not reveal an association in others.

More studies must be implemented to elaborate on this limited scope.

## Introduction and background

Background

Parturition is an especially vulnerable time for the subsequent onset of severe psychotic symptoms in women with no prior psychiatric history. The postpartum (PP) period, in particular, may include transformational changes in a female’s behavioral health. The exact etiology of first-onset postpartum psychosis is unknown. Recent evidence suggests, however, that postpartum psychotic episodes in women with no prior psychiatric history may be associated with traumatic childbirth experiences as a result of periparturient complications. These may include excessive antepartum hemorrhage, excessive postpartum hemorrhage, eclampsia, pre-eclampsia, cesarean section, sleep deprivation, puerperal sepsis, concurrent coronavirus disease 2019 (COVID-19) infection, birth canal injuries, placental abruption, and uterine rupture. With an awareness and understanding of the risk factors associated with first-onset postpartum psychosis, the management of this disease may be facilitated to achieve enhanced outcomes.

Introduction

Approximately half of women affected by postpartum psychosis (PPP) have no history of psychiatric illness, revealing the peri-parturition period to be of particularly high risk for the onset of severe psychiatric symptoms when compared to nulliparous or pregnant states [1]. It is well-documented that there is an increased risk for the initial start of acute psychotic episodes in the initial weeks after parturition, especially when compared with the risk of occurrence outside the postpartum period [2-5]. Epidemiological research replicated over decades indicates that approximately one in 1000 women experiences postpartum psychosis as a first-onset psychiatric illness with no prior psychiatric incident [6,7]. Although the American Psychiatric Association’s Diagnostic Statistical Manual of Mental Disorders (DSM-V) and the World Health Organization’s ICD-10 Classification of Mental and Behavioral Disorders also classifies puerperal psychosis as a feature of bipolar and related disorders, as well as a possible characteristic of the schizophrenia spectrum, the scope of this paper will be limited to a discussion of the women with a diagnosis of brief psychotic disorder with postpartum onset, particularly with no history of psychiatric diagnoses [8].

The most essential feature of postpartum psychosis is a disturbance that involves the sudden onset of at least one of the following positive psychotic symptoms: delusions, hallucinations, disorganized speech (e.g., incoherence), or grossly abnormal psychomotor behavior, including catatonia (Criterion A). Sudden onset is defined as a change from a nonpsychotic state to a psychotic state within two weeks. An episode of the disturbance lasts at least one day but less than one month, and the individual eventually has a full return to the premorbid level of functioning (Criterion B). Criterion C indicates that the disturbance is not better explained by a depressive or bipolar disorder with psychotic features, schizoaffective disorder, or schizophrenia. It is further not attributable to the physiological effects of a substance (e.g., a hallucinogen) or another medical condition (e.g., subdural hematoma) [9]. In addition to the five symptom domain areas identified in the diagnostic criteria, the assessment of cognition, depression, and mania symptom domains is vital for making critically important distinctions between the various schizophrenia spectrum and other psychotic disorders.

A deeper understanding of the underlying etiologies and risk factors of first-onset postpartum psychosis will facilitate both the prevention and treatment of postpartum psychiatric episodes and significantly improve patient outcomes. The mechanism of onset perhaps may be related to specific physiological changes at birth in predisposed women. Periparturient complications, including sleep deprivation, have been described as a possible causal trigger for postpartum psychosis [8]. Although the precise underlying mechanisms of first onset postpartum psychosis have remained elusive, this systematic review will examine the association of postpartum psychosis in women with no psychiatric history and periparturient complications, including excessive antepartum hemorrhage, excessive postpartum hemorrhage, eclampsia, pre-eclampsia, cesarean section, sleep deprivation, puerperal sepsis, concurrent COVID-19 infection, birth canal injuries, placental abruption, and uterine rupture.

## Review

Methods

Search Strategy for the Identification of Studies and Information Sources

**Figure 1:**
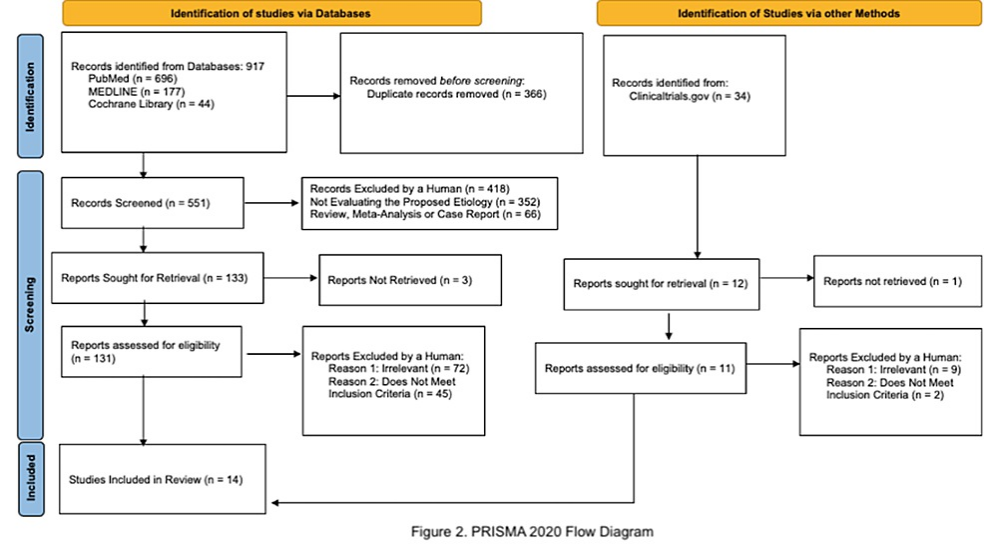
PRISMA 2020 Flow Diagram PRISMA: Preferred Reporting Items for Systematic Reviews and Meta-Analyses

In accordance with the 2021 Preferred Reporting Items for Systematic Reviews and Meta-Analyses (PRISMA) guidelines, a search strategy to retrieve references relating to postpartum psychosis perinatal risk factors was implemented using the following keywords: “postpartum psychosis”, “postpartum psychosis etiology”, and "postpartum psychosis risk factors" in the following databases: PubMed, MEDLINE, and Cochrane Library, as well as clinicaltrials.gov. Searches were conducted on April 5, 2022, from inception, and repeated on June 12, 2022, in order to preclude omitting newly published articles. Additionally, research faculty at Florida International University Robert Stempel College of Public Health School were contacted in order to confirm that unpublished articles were not overlooked. Searches were also not limited to any language. Table 1 (systemic review protocol) and Figure 1 (PRISMA flow diagram) elucidate the search strategy and selection of inclusion criteria.

**Table 1:** Systemic Review Protocol

Search	Items Found
PubMed: Inception to April 3, 2022	
“Postpartum Psychosis” AND “Etiology”	462
“Postpartum Psychosis” AND “Risk Factor”	234
MEDLINE: Inception to April 3, 2022	
“Postpartum Psychosis” AND “Etiology”	92
“Postpartum Psychosis” AND “Risk Factor”	85
Cochrane Library: Inception to April 3, 2022	
“Postpartum Psychosis”	44
Clinicaltrials.gov: Inception to April 3, 2022	
Condition: “Postpartum Psychosis” and Other Term “Risk Factor”	34
Condition: “Postpartum Psychosis” and Recruitment “Completed”	12
Condition: “Postpartum Psychosis” and Recruitment “Completed” and Study Results “Available”	12

Eligibility Criteria and Selection Process

At the outset of the search process, 585 articles were collected following the exclusion of duplicate articles from the databases and website. The article abstracts and article titles of the identified publications were screened independently by all seven authors and studies were selected if they met the following inclusion criteria:

(1) Patients were diagnosed with postpartum psychosis per the guidelines in the American Psychiatric Association’s Diagnostic and Statistical Manual of Mental Disorders (DSM-V), DSM-IV, or World Health Organization’s ICD-10 Classification of Mental and Behavioral Disorders;

(2) At least one patient included in the study presented with no prior psychiatric diagnoses, hospitalizations, or history;

(3) The study evaluated the correlation of periparturient etiologies to first-onset postpartum psychosis;

(4) Studies could not be reviews, systemic reviews, or meta-analyses.

Next, 131 full articles were obtained for the selected abstracts/titles and were independently read and annotated by all seven authors. Studies were further excluded if they did not meet the inclusion criteria, if they were unable to be retrieved (four articles), or if the article was not relevant to the proposed etiology of postpartum psychosis. All articles were unanimously agreed upon by all seven authors. Moreover, the seven authors were not blinded from the names of the journals that published the studies, the authors of the articles, the results of the articles, or the studies’ discussion.

Data Collection and Data Items

Significant findings of each study were qualitatively recorded by all seven authors using a data extraction template in order to synthesize information. The methodology for data extraction was adapted from Dr. Tianjing Li, Assistant Professor at John’s Hopkins Bloomberg School of Public Health [10]. Ultimately, 14 studies meeting the inclusion and exclusion criteria were utilized for systemic review.

The following outcome measures were considered:

(1) Association of periparturient risk factors with physician-reported postpartum psychosis rating scales;

(2) Association of periparturient risk factors with patient-reported postpartum psychosis rating scales (for example, the self-reporting Prodromal Questionnaire, PQ-16, derived from the 92-item Prodromal Questionnaire (PQ-92) used by clinicians to rapidly screen for postpartum psychosis;

Results

Study Characteristics and Risk of Bias

Upon examination, obstetric delivery methods concluded a positive association between emergency cesarean sections and first-onset postpartum psychosis in two studies (Table 2). Peripartum complications, such as excessive antepartum or postpartum hemorrhage, pre-eclampsia, concurrent COVID-19 infection, birth canal injuries, placental abruption, uterine rupture, and stillbirth results in a positive correlation with postpartum psychosis. Sleep deprivation and disruption of circadian rhythms also had studies that did and did not exhibit a positive association with postpartum psychosis. There was one study that highlighted a positive correlation with first-time mothers having more sleep deprivation than multiparous women, which could contribute to the development of postpartum psychosis, however, some of these women may or may not have had a past psychiatric history (Table 2). Included were contradicting articles regarding the positive association between first-onset postpartum psychosis and peripartum complications (at least one study shows no correlation) (Table 2).

**Table 2:** Characteristics of Included Studies for the Systematic Review PQ: Prodromal Questionnaire; PTSD: post-traumatic stress disorder; PMH: past medical history; DSM: American Psychiatric Association’s Diagnostic and Statistical Manual of Mental Disorders; PP: postpartum; PPE: postpartum episode; NPPE: non-postpartum episode; HC: healthy controls; PPD: postpartum depression; Asx: asymptomatic; SES: socioeconomic status

Study	Study Type	Outcome	Risk of Bias
Adjorlolo S, Mensah G, Badzi C. 2022. [11]	Cross-Sectional Study	After self-reported data collection via PQ-16 and PQ-9, the Bonferroni post hoc test revealed high-risk psychosis groups (n = 702) reported significantly greater COVID-19 concerns and sleep difficulty than the No/Low-risk group. χ2 = 35.10, p < 0.001. Exclusion criteria for all patients included a history of a mental health disorder.	Cross-sectional study design limits establishing causality; Selection bias due to recruitment of participants with high school education. Recall bias of participants (self-reported symptoms).
Antoniou E, Eirini O, Kassiani P, Alexandros P et al. 2021. [12]	Case Report	A patient with undiagnosed psychiatric history (later diagnosed with bipolar disorder and PTSD) presented with an emergency C-section due to pre-eclampsia, which served as a catalyst for the patient's PPP.	n=1 important limitation; anecdotal nature of case report due to conflicting nature of PMH
Bågedahl-Strindlund, M. 1986. [13]	Cohort	incidence was 1.2 per 1000 live births (42 cases per 36,588 live births), peaked within 3 months; discord and peripartum stress were associated with PP.	Confounding variables: n=39 in total,but exclude n=7 schizophrenia Dx.1986 study not replicated.
Bergink, V., Burgerhout, K.M. Weigelt, K., Pop, V.J et al. 2013. [14]	Prospective Cohort	Decreased circulating T cells/GR elevated monocytes and CCL2, implicated in peripartum complications indicate a positive association between immune-mediated complications of delivery with PP	n=63; confounding variables
Bergink, V., Laursen, T. M., Johannsen, B. M. W., Kushner, S. A., Meltzer-Brody, S., & Munk-Olsen, T. 2015. [4]	Cohort	Preeclampsia in first onset PPP positive association: Out of the 2723 women with psychiatric episodes during the first 360 days postpartum, 162 had pre-eclampsia during pregnancy (5.93%). Among the 39.8% (n = 1087) of women who received their psychiatric diagnosis during the first 3 months postpartum, 71 had pre-eclampsia during pregnancy (6.53%).	Selection Bias, Limited Scope. No replication of results.
Franchi et al. 2020. [15]	Case Report	35 weeks gestation COVID-19 diagnosis → emergency C-section due to COVID complications' fetal distress → PP association	N=1; Case report bias
Fusté, M., Pauls, A., Worker, A., Reinders, A. A. T. S., Simmons, A., Williams, S. C. R., … Dazzan, P. 2017. [16]	Cross-Sectional	Women with PPE showed smaller anterior cingulate gyrus, superior temporal gyrus, and parahippocampal gyrus compared to NPPE women. These regions also showed decreased surface area. Moreover, the NPPE group showed a larger superior and inferior frontal gyrus volume than the HC.	N=9 for patients with no psychiatric history, therefore very small statistical power that may be confounded with the inclusion of schizophrenic patients. Findings need to be validated in larger studies.
Kendall, R. et al. 1981. [17]	Cohort	C-section positively associated with PP	40-year-old study that has not been widely reproduced.
Langan Martin J., McLean G, Cantwell R, Smith D et al. 2016. [1]	Cohort	Admissions during the postpartum period were elevated in primiparous women (relative to multiparous) after controlling for social deprivation and age; the risk of admission due to psychosis was highest in the second week following childbirth complications; 3290 pregnancy-related psychiatric admissions were assessed.	Epidemiological study
Lewkowitz et al. 2019. [18]	Retrospective Cohort	The stillbirth of an infant was associated with a 2.5 times increased risk of severe psychiatric disorder within the first postpartum year	Confounded by including women with PMH psychiatric disorders
Meltzer-Brody, S., Maegbaek, M. L., Medland, S. E., Miller, W. C., Sullivan, P., & Munk-Olsen, T. 2017. [19]	Cohort	PPD and postpartum acute stress reactions were NOT associated with pregnancy and obstetrical complications. For PPD, hyperemesis gravidarum (IRR 2.69, 95% confidence interval (CI) 1.93–3.73), gestational hypertension (IRR 1.84, 95% CI 1.33–2.55), pre-eclampsia (IRR 1.45, 95% CI 1.14–1.84) and cesarean section (C-section) (IRR 1.32, 95% CI 1.13–1.53) were associated with increased risk. For postpartum acute stress, hyperemesis gravidarum (IRR 1.93, 95% CI 1.38–2.71), preterm birth (IRR 1.51, 95% CI 1.30–1.75), gestational diabetes (IRR 1.42, 95% CI 1.03–1.97), and C-section (IRR 1.36, 95% CI 1.20–1.55) were associated with increased risk. In contrast, the risk of PP was not associated with pregnancy or obstetrical complications.	Population cohort study with confounding variables
Nager A.; K. Sundquist; V. Ramírez-León; L. M. Johansson. 2008. [20]	Cohort	Positive association with emergency “acute” C-section and PPP; n=1413.	Demographic and sociological limitations (only first-time mothers included; inclusion of mothers with psychiatric history confounds
Sanchez, S. E., Friedman, L. E., Rondon, M. B., Drake, C. L., Williams, M. A., & Gelaye, B. 2020. [21]	Cross-sectional Cohort	2,068 participants eligible for the study → excluded 6 27.6% were assessed as having a high risk of psychosis; Stress-related sleep disturbances during pregnancy are associated with increased odds of psychiatric disorders.	Limited scope in PP; recall bias due to self-reported PQ-16; confounding
Sharma et al. 2004. [22]	Cohort	The majority of women (60%) suffered from DSM-IV bipolar or schizoaffective- bipolar type disorders; sleep loss resulting from nighttime delivery is implicated in the etiology of PPP; first-time mothers are more likely to be affected by sleep changes in the postpartum period in comparison to multiparous women (57% of all women in the postpartum psychosis group were primiparous.); the first episode of PPP was more likely to have a history of obstetric complications as compared to normal comparison subjects; obstetric complications included cesarean section, incubator/blue/resuscitation, nuchal cord, and abnormal gestational age.	Confounding due to the inclusion of women with a previous psychiatric history
Subramanyam, A. A., Nachane, H. B., Mahajan, N. N., Shinde, S., D Mahale, S., & Gajbhiye, R. K. 2020. [23]	Case Series	3rd Trimester COVID coinciding with complex delivery → Possible risk factor for PPP; 3 women diagnosed w PPP associated with Asx COVID (2 C-sections)	Very small n=4. Confounding factors with little information on COVID-19
Upadhyaya, S., Sharma, A., & Raval, C. 2014. [24]	Cross-Sectional Case Control	Positive correlation of PP with perinatal maternal complications	n=100; confounding due to PMH psychiatric diagnoses in some patients
Videbech, P., & Gouliaev, G. 1995. [25]	Case Control	Greater incidence of PPP in single mothers, lower SES, and fewer resources.	Not replicated since published; limited n

## Conclusions

Postpartum psychosis affects approximately one in 500 mothers after giving birth, and a prior psychiatric diagnosis, such as bipolar, increases the risk in pregnant women. In order to narrow risk factors, only patients without a prior psychiatric history were used in the study, as prior psychiatric history is already a well-known risk factor but not the cause. The etiology of first-onset postpartum psychosis in women with no previous psychiatric history is complex and likely the result of a culmination of several factors. These factors include but are not limited to excessive antepartum hemorrhage, postpartum hemorrhage, eclampsia, pre-eclampsia, cesarean section, sleep deprivation, puerperal sepsis, concurrent COVID-19 infection, birth canal injuries, placental abruption, and uterine rupture. Further, mothers experience extraordinary physiological and social changes during parturition, not just the well-studied sudden hormonal changes. In investigating the relationship between first onset postpartum psychosis and several peripartum complications, individual cohort studies, case-control studies, and case reports conflict and offer little consensus.

Further understanding of the underlying etiologies and risk factors of first-onset postpartum psychosis is needed in order to facilitate the prevention and treatment of postpartum psychiatric episodes. Many of the studies that showed a positive correlation lacked statistical power, and future research should focus on the possible positive correlations using larger numbers of women in order to cultivate predictive risk assessments for vulnerable patients. In addition, a better understanding of the physiological and social changes in regard to first-onset postpartum psychosis could also benefit treatment and management.
